# Crizotinib in anaplastic lymphoma kinase**-**positive anaplastic large cell lymphoma in the setting of renal insufficiency: a case report

**DOI:** 10.1186/s13256-016-0963-y

**Published:** 2016-06-14

**Authors:** Shalin Kothari, Najam Ud-Din, Michele Lisi, Thomas Coyle

**Affiliations:** 1 Department of Medicine, SUNY Upstate Medical University, 750 E Adams St., Syracuse, NY 13210 USA; 2 Division of Hematology/Oncology, Department of Medicine, SUNY Upstate Medical University, 750 E Adams St., Syracuse, NY 13210 USA; 3 Department of Radiology, SUNY Upstate Medical University, 750 E Adams St., Syracuse, NY 13210 USA

**Keywords:** ALK, Anaplastic large cell lymphoma, Crizotinib, Renal insufficiency

## Abstract

**Background:**

*In vitro* studies confirmed cytoreductive anti-tumor activity of crizotinib in experimental models of anaplastic large cell lymphoma in 2007. One case series and a few case reports describe the use of crizotinib in relapsed or refractory anaplastic lymphoma kinase-positive anaplastic large cell lymphoma. Even though data are limited regarding the dose of crizotinib in renal insufficiency, our case was successfully treated with a lower dose of crizotinib.

**Case presentation:**

We report the case of a 48-year-old white man who had progressive disease after three prior cycles of cyclophosphamide, doxorubicin, vincristine and prednisone and three cycles of ifosfamide, carboplatin, and etoposide, and was not a candidate for high-dose chemotherapy and transplant due to poor performance status and renal insufficiency; he had a complete and durable response to single agent crizotinib. Crizotinib was given at a reduced dose (250 mg once daily) due to his renal insufficiency. He has been in complete remission for more than 2 years.

**Conclusions:**

Our experience confirms the activity of crizotinib in this disease; it suggests that long-term treatment with crizotinib is a reasonable option in patients who are not candidates for more aggressive therapy and indicates that crizotinib can be used successfully at reduced doses in patients with pre-existing renal insufficiency. The role and timing of crizotinib in anaplastic lymphoma kinase-positive anaplastic large cell lymphoma is unclear, but the current literature that we review here provides promising results that may lead to studies of crizotinib earlier in the course of disease.

## Background

Systemic anaplastic large cell lymphoma (ALCL) is a rare subtype of peripheral T cell lymphoma representing approximately 3 % of non-Hodgkin’s lymphoma (NHL), often presenting with advanced disease, B symptoms and extranodal disease [1]. Anaplastic lymphoma kinase-positive (ALK+) ALCL is characterized by a specific chromosomal translocation, t(2;5)(p23;35) which fuses the *ALK* gene on chromosome 2 with the *nucleophosmin* (*NPM*) gene on chromosome 5, resulting in a NPM-ALK fusion protein, ALK overexpression and constitutive tyrosine kinase activity [2]. Other partner genes for *ALK* translocation events have been described, including *TPM3*, *TFG*, *MSN*, *CLTC*, and *ATIC*. However, NPM-ALK accounts for more than 75 % of the ALK+ ALCL cases reported [3, 4]. ALK+ ALCL generally have a good response to standard chemotherapy and relatively good prognosis, with approximately 60 % of patients remaining in remission 5 years after frontline therapy [5]. However, the prognosis of patients with refractory or relapsed disease is poor [6]. Furthermore, the small cell variant of ALK+ ALCL has a worse prognosis [7]. Approaches to treatment of relapsed or refractory disease have included high-dose chemotherapy and autologous stem cell transplant (auto-SCT), allogeneic stem cell transplant (allo-SCT), and a number of second-line chemotherapy agents including brentuximab vedotin [8]. Recently, several case reports and small series have shown impressive responses of relapsed/refractory ALK+ ALCL to crizotinib, a specific inhibitor of the ALK kinase. We now report further a case of a patient with refractory ALK+ ALCL with a complete durable response to single agent crizotinib. This patient has been in (CR) for more than 2 years, on a reduced dose of crizotinib due to pre-existing renal failure.

## Case presentation

Our patient was a 48-year-old white man with past medical history of diabetes mellitus type 2 who was diagnosed with stage IV ALK+ ALCL, small cell variant, after presenting with B symptoms, right axillary and supraclavicular lymphadenopathy and splenomegaly. ALK positivity was confirmed using immunohistochemistry and fluorescent *in situ* hybridization employing an ALK break-apart probe. He received three cycles of cyclophosphamide, doxorubicin, vincristine, and prednisolone (CHOP) but had progression of disease with a necrotic spleen, continued B symptoms, and a malignant left pleural effusion. He was subsequently treated with splenectomy and drainage of the pleural effusion with a PleurX catheter, and his chemotherapy was changed to ifosfamide, carboplatin, and etoposide (ICE) in an attempt to prepare for an auto-SCT. The pathology from his spleen showed persistent viable lymphoma. He received three cycles of ICE chemotherapy. He initially partially responded to it, but the therapy was complicated by episodes of encephalopathy due to ifosfamide and the development of progressive renal insufficiency. His baseline serum creatinine was 0.8 mg/dl, but it rose to 1.8 mg/dl by the time of the third cycle of ICE and subsequently peaked at a level of 5 to 6 mg/dl 2 months later. A renal biopsy showed lymphocytic interstitial nephritis.

**Fig. 1:**
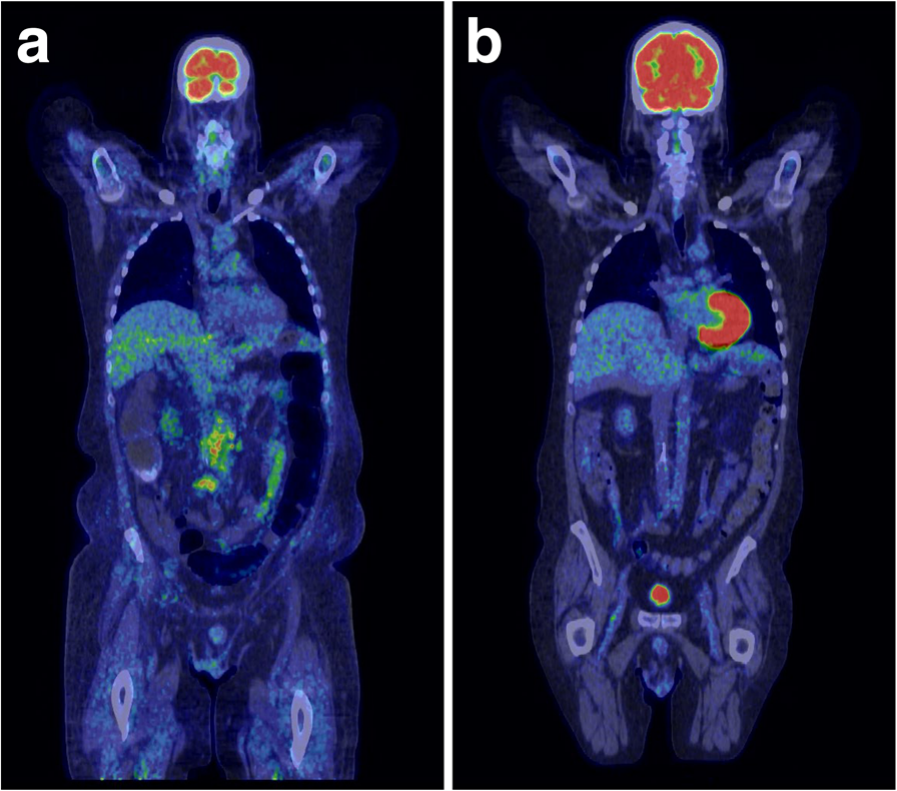
**a** ^18^F-fluorodeoxyglucose positron emission tomography/computed tomography on Day 0 shows significant retroperitoneal lymphadenopathy. **b** ^18^F-fluorodeoxyglucose positron emission tomography/computed tomography on Day 76 shows complete resolution of the retroperitoneal lymphadenopathy

Crizotinib, at a dose of 250 mg twice a day, was added to the regimen immediately following the second cycle of ICE, but it was discontinued after 7 days due to diarrhea. Six weeks after receiving his third cycle of ICE, he again developed fevers (temperatures >39 °C), axillary adenopathy, and a decline in Eastern Cooperative Group (ECOG) performance status from 1 to 3. Persistent disease was demonstrated by ^18^F-fluorodeoxyglucose (FDG) avidity on positron emission tomographic (PET) and computed tomographic (CT) images of his retroperitoneal and right axillary lymph nodes. We thought that he was no longer a candidate for auto-SCT or allo-SCT due to a poor performance status, resistant disease, and renal failure. Crizotinib was restarted at a dose of 250 mg once a day (Day 0). His fevers disappeared within 24 hours. Other B symptoms and palpable axillary adenopathy resolved in a week. PET and CT images performed at Day 76 showed complete resolution of previously FDG-avid lesions (Fig. 1). His CR has persisted for 29 months on continued therapy with 250 mg of crizotinib orally once daily. Adverse effects including transient thrombocytopenia and diarrhea have been mild. His renal disease has stabilized with creatinine levels between 4 mg/dl and 5 mg/dl without specific treatment and without the need for dialysis.

Crizotinib is a well-tolerated small molecule inhibitor of the ALK tyrosine kinase. It has significant activity in non-small cell lung cancers (NSCLCs) bearing an activating *EML4-ALK* translocation and is approved by the US Food and Drug Administration (FDA) for this indication. Crizotinib has been shown to have *in vitro* activity against ALK-positive lymphomas [9]. Crizotinib induces apoptosis due to down-regulation of pSTAT3 and BCL-2 family proteins [10] and has excellent potential to treat patients with refractory ALK-positive ALCL, such as our case.

Several case reports and small series have appeared on the use of crizotinib in adult patients with relapsed/refractory ALK+ ALCL which indicate that such lymphomas have a high chance of responding to crizotinib, even when heavily pretreated, with approximately half enjoying long-lasting responses; however, no pretreatment parameter is able to predict a durable CR [11–14]. These cases are summarized in Table 1. A high percentage of patients, but not all, respond to treatment with prompt resolution of B symptoms and rapid complete radiographic responses as shown by PET-CT. The crizotinib dose generally used was 250 mg twice a day. The treatment was well tolerated. In a number of the cases, crizotinib was used as a bridge to allo-SCT, although in one of those studies the patient relapsed in 4 weeks on crizotinib before the allo-SCT could be done [12]. Other patients have continued on treatment with single agent crizotinib with durable response. In the largest of these series, reported by Gambacorti Passerini *et al*., crizotinib was given to nine patients with refractory/relapsed ALK+ ALCL and all of them responded initially. Four of these patients remained in CR on continuous crizotinib therapy at 21 to 40 months follow-up. Two had undergone allo-SCT and remain in CR, of which one was still on crizotinib. Two of the nine patients had subsequent progression of the disease [11]. A prospective phase I trial in pediatric patients with several malignancies was performed by the Children’s Oncology Group. It showed that of nine patients with ALK-mutated NHL, eight responded and seven had CRs and five remained on crizotinib in long-term remission [15].

**Table 1:** Reported cases of the use of crizotinib in anaplastic lymphoma kinase-positive anaplastic large cell lymphoma

Study	Age	Stage (Ann Arbor)	ECOG	Previous therapy lines	Response, in months
Gambacorti Passerini *et al*. 2014 [11]	26	IIIB	2	CHOP, DHAP, HD-VP16	CR, >40
	19	IVB	3	CHOP, DHAP, BEAM	CR, 2
	22	IIB	1	CHOP, VAD, H-CyVAD	CR, >35
	20	IIB	2	CHOP, DHAP, BEAM	CR, 2
	47	IIIB	2	IEV, CHOP, DHAP	CR, >30
	28	IIIBe	2	CHOP, DHAP, miniBEAM	CR, 2
	34	IVBe	2	CHOP, ESHAP	CR, 3
	38	IVB	4	CHOP, DHAP, VIM	CR, 8
	55	IIIB	1	CHOP	CR, >21
Ordemann *et al*. 2013 [12]	29	na	na	CHOP-21, DHAP, Dexa-BEAM	PR, 1
Cleary *et al*. 2014 [13]	34	na	na	CHOP, gemcitabine-based therapy, pralatrexate, Mtx, brentuximab	CR, 30; allo-SCT at week 13
Conyers *et al*. 2014 [14]	22	IIIB	na	CHOP	CR, >21; allo-SCT at 2 months
Current case	48	IV	3	CHOP, ICE	CR, >29

*allo-SCT* allogeneic stem cell transplant*, BEAM* carmustine, etoposide, cytarabine, melphalan, *CHOP* cyclophosphamide, doxorubicin (adriamycin), vincristine, prednisone, *CR* complete response, *Dexa-BEAM* dexamethasone, carmustine, etoposide, cytarabine, melphalan, *DHAP* dexamethasone, cisplatin, cytarabine, *ECOG* Eastern Cooperative Group, *ESHAP* etoposide, methylprednisolone, cytarabine, cisplatin, *H-CyVAD* alternate regimens of 1) cyclophosphamide, vincristine, doxorubicin (adriamycin), dexamethasone; 2) methotrexate and cytarabine; *HD-VP16* high-dose etoposide, *ICE* ifosfamide, carboplatin, etoposide, *IEV* Ifosfamide, epirubicin, etoposide, *miniBEAM* carmustine, etoposide, cytarabine, melphalan, *Mtx* methotrexate, *na* not available, *PR* partial response, *VAD* vincristine, doxorubicin, high-dose dexamethasone, *VIM* ifosfamide, mitoxantrone, etoposide

Our patient did not tolerate crizotinib given at full dose in combination with ICE chemotherapy. It is unclear if this was due to concomitant ICE chemotherapy or due to decreased clearance of the drug due to his renal insufficiency. Crizotinib was restarted at a reduced dose of 250 mg daily as a single agent after recovery from the last cycle of ICE chemotherapy. He has tolerated this without problems and without further kidney injury or the need for dialysis. Renal pathology had showed acute interstitial nephritis, which was thought to be secondary to proton pump inhibitor (PPI) that was initiated around that time and the PPI was stopped immediately. At the time of initiation of reduced dose of crizotinib, no literature was available on the interaction of crizotinib with reduced renal function. In NSCLC trials, crizotinib at 250 mg twice a day reaches steady state in 15 days and then dose levels decrease non-linearly. There were no differences in pharmacokinetics (PK) levels for mild to moderate renal insufficiency. It would be a good study question to look at the PK levels for crizotinib in patients with renal impairment in conditions other than NSCLC. Recent literature recommends arbitrary dose adjustment (200 mg twice a day or 250 mg once daily) in the presence of renal insufficiency [16], but the exact dosing in this situation still remains unknown and further research is needed.

## Conclusions

This experience suggests that single agent crizotinib is a viable option for patients with relapsed/refractory ALK+ ALCL who are not candidates for high-dose chemotherapy and auto-SCT or allo-SCT. Since crizotinib can yield long-term remissions in patients with relapsed/refractory ALK+ ALCL, it is unclear if all patients with relapsed/refractory disease require high-dose chemotherapy and allo-SCT even if they are candidates for it. In addition, this experience demonstrated that crizotinib could be successfully employed at reduced doses in patients with renal insufficiency.
